# A Neglected Case of a Benign Phyllodes Tumor Presenting as a 10.5 kg Breast Mass

**DOI:** 10.7759/cureus.75230

**Published:** 2024-12-06

**Authors:** Neyaz Ahmad, Krishna Murari, Zenith Kerketta, Khushboo Rani, Anish Baxla, Sunny

**Affiliations:** 1 General Surgery, Rajendra Institute of Medical Sciences, Ranchi, IND

**Keywords:** benign breast lesions, breast benign and malignant surgery, giant phyllodes tumour, large benign phyllodes, neglected tumors

## Abstract

Phyllodes tumor is a type of fibroepithelial neoplasm involving the breast. This tumor is rarely reported in adolescents and the elderly and has a peak incidence in middle-aged women. Histologically, phyllodes tumors are classified as benign, borderline, or malignant. Phyllodes tumor though rare can mimic breast carcinoma and can grow rapidly to massive size. Hence, carcinoma breast should always be kept as a differential diagnosis. Phyllodes tumors can present in different sizes and consistency. Giant forms can lead to postural deformities and back pain. There can be associated skin changes and ulceration due to pressure necrosis. Core needle biopsy is mandatory for the confirmation of diagnosis. It helps in decision-making for further management of the tumor. There is always a risk of recurrence in these tumors. An adequate resection margin is required to avoid any chances of relapse.

Most of the phyllodes tumors do not have lymph node metastases and they rarely spread. However early and prompt surgical management is needed to avoid any possibility of malignant transformation. Here we report a neglected case of phyllodes tumor in a 68-year-old tribal woman with a huge left breast mass for 12 years.

## Introduction

Phyllodes tumors are infrequently seen fibroepithelial tumors that constitute 0.3-1% of all breast tumors [1]. Phyllodes tumors are also known as cystosarcoma phyllodes as they have a fleshy appearance and a tendency to contain macroscopic cysts. The term “phyllodes,” which means leaf-like, describes the typical papillary projections that are seen on pathologic examination. As per WHO, these tumors range histologically in a spectrum from benign to malignant based on the degree of stromal cellular atypia, mitotic activity per 10 high power fields, and degree of stromal overgrowth [2]. The majority of the tumors are benign (60-75%). Borderline and malignant types share 15-20% and 10-20% of all cases [3]. These tumors are typically seen in women with ages ranging from 35 to 55 years, nearly about 20 years later than fibroadenoma [4]. Phyllodes tumors have a median size of about 4 cm. 20% of these tumors can grow larger than 10 cm known as giant phyllodes tumors. Axillary lymph nodes can be palpated in up to 10-15% of patients, but only a few (<1%) had pathological positive nodes [5]. Noguchi et al. showed that in certain cases of fibroadenomas, a somatic gene mutation results in a monoclonal proliferation, and they are histologically similar to the polyclonal element but with a propensity to local recurrence and progression to a phyllodes tumor [6]. Preoperative histological diagnosis in cases of phyllodes is important as it guides us through further management and prevention of recurrences. Mammography and ultrasonography (USG) are the most preferred tools for routine imaging of breast lumps. Wurdinger et al. explained phyllodes tumors have round or lobulated shapes with well-defined margins, heterogenous internal structures, and non-enhancing internal structures [7]. These features help in differentiating phyllodes tumor from fibroadenoma. Core needle biopsy is preferred over fine-needle aspiration cytology (FNAC) as it provides extra architectural information. Komenaka et al. found that the core needle biopsy has a sensitivity of 99% and negative predictive value and positive predictive value of 93% and 83%, respectively, for the diagnosis [8]. As per recent guidelines, wide local excision means excision with surgical margins ≥1 cm. Narrow surgical margins have a higher risk of local recurrence risk, particularly in borderline and malignant types of phyllodes tumors [9]. Here, we present a neglected case of a 10.5 kg benign phyllodes tumor in a 68-year-old female.

## Case presentation

**Figure 1:**
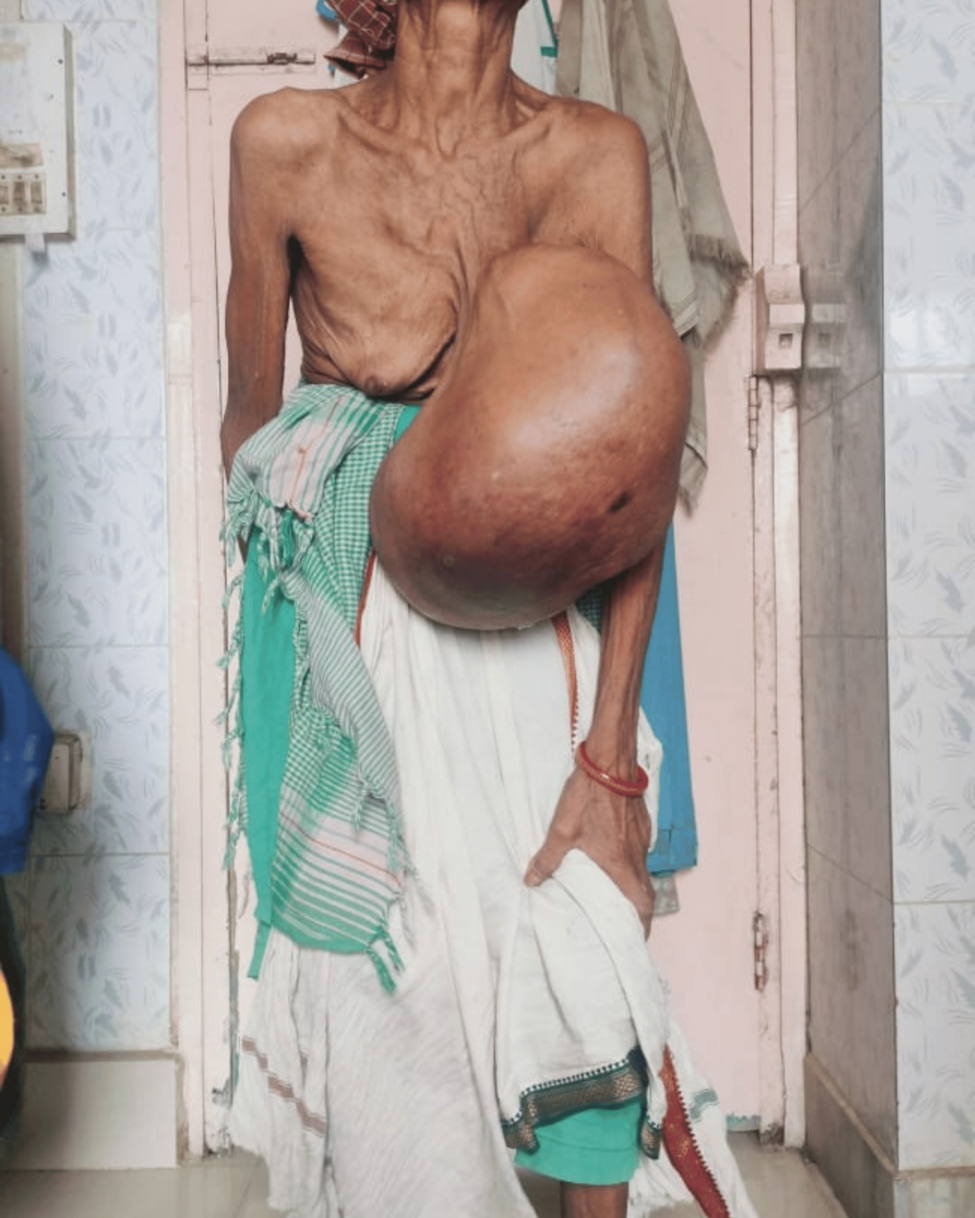
Woman presenting with a huge left breast mass (anterior view)

**Figure 2:**
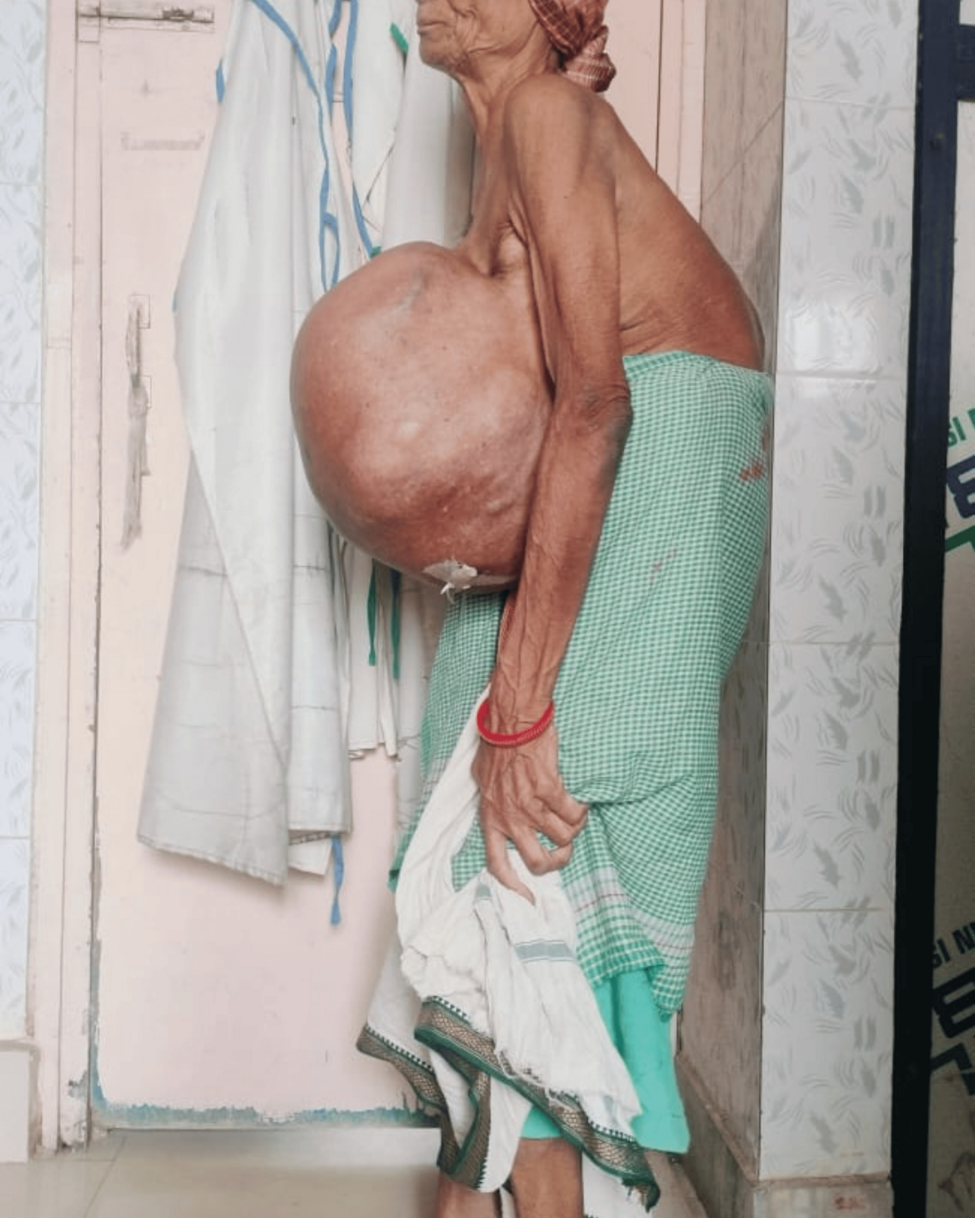
The patient presented with gross kyphosis due to the weight of the tumor

A 68-year-old tribal woman presented to the emergency room with a huge left breast mass for 12 years (Figure 1). The patient had postural deformities and difficulty even standing in an erect position due to the weight of the tumor (Figure 2). It was initially the size of a lemon, which progressed to the size of a basketball over 10 years. There was an accelerated increase in size for the past two years, with variations in consistency and skin changes. The patient was forced to use the help of other family members to perform routine day-to-day activities. It was associated with obvious back pain and fatigue.

Examination

There was a massively enlarged left breast on physical examination with dimensions 36x24x20 cm. Inspection and palpation revealed a tense, shiny, tender left breast mass with variable consistency. The left breast was mobile and was not fixed to the underlying chest wall. The skin over the swelling showed discoloration, and the nipple-areolar complex was unremarkable, with a severely retracted nipple. There were no signs of any axillary lymphadenopathy or clinical evidence of any metastases. The patient had kyphosis and scoliosis due to the weight of the tumor. The contralateral breast and nipple areolar complex was normal concerning her age. The patient had severe pallor on general examination.

**Figure 3:**
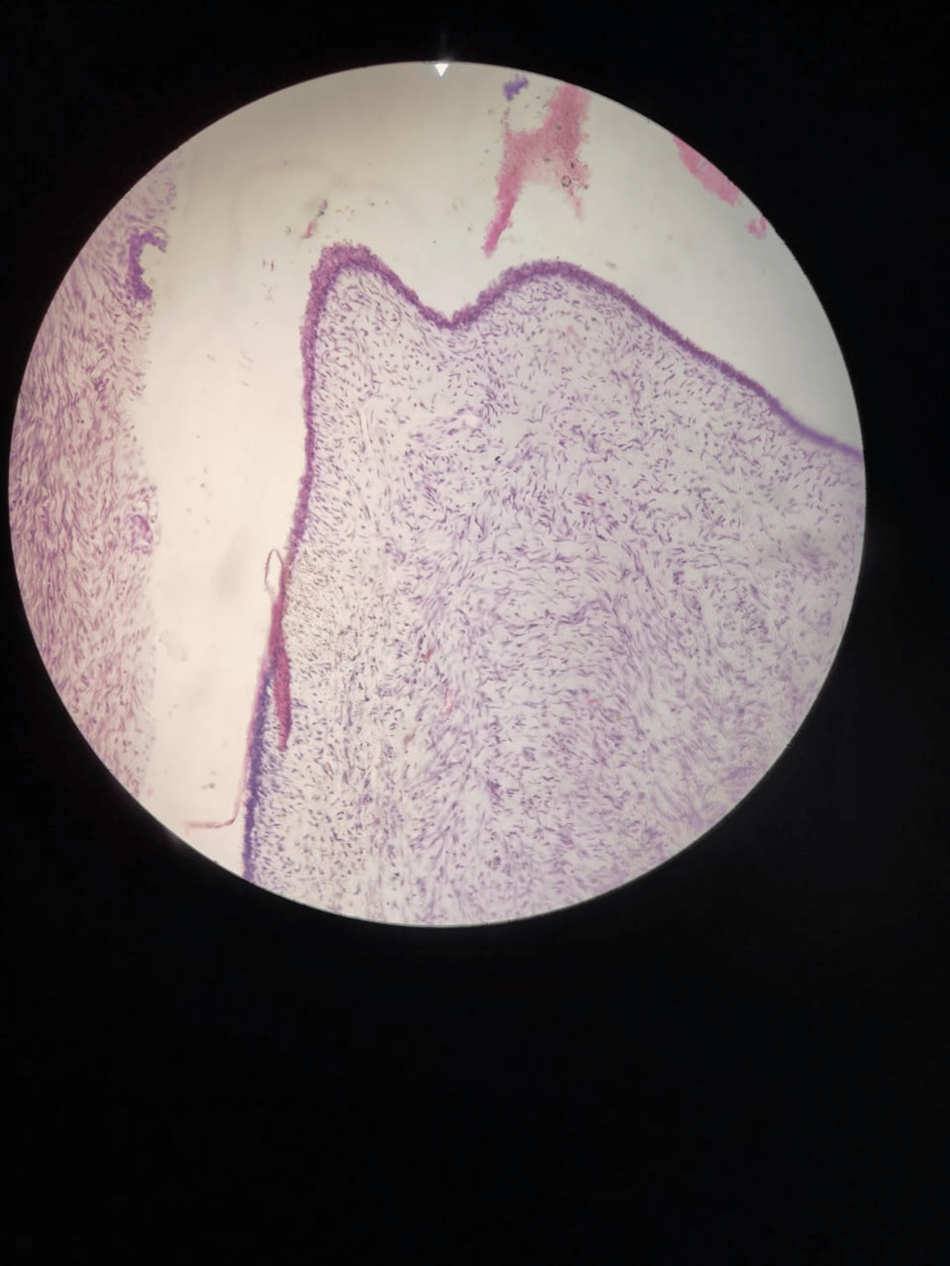
Marked stromal proliferation in the background of normal ductal epithelium

Routine blood investigations and USG of bilateral breast and axilla were ordered. The blood picture revealed severe anemia with hemoglobin of 7g/dL. Renal function and liver function tests were within normal limits. USG bilateral breast and axilla revealed a large heterogenous solid lesion of the left breast with internal thick septations, cystic component, and microcalcification. There was marked internal vascularity on Doppler. The ipsilateral axilla could not be assessed due to an inadequate field of view. X-ray of the chest showed intense calcification on the left side, causing obscuration of left lung parenchyma. FNAC and Tru-cut biopsy suggested it to be a benign phyllodes tumor with marked stromal proliferation (Figure 3).

Surgery

**Figure 4:**
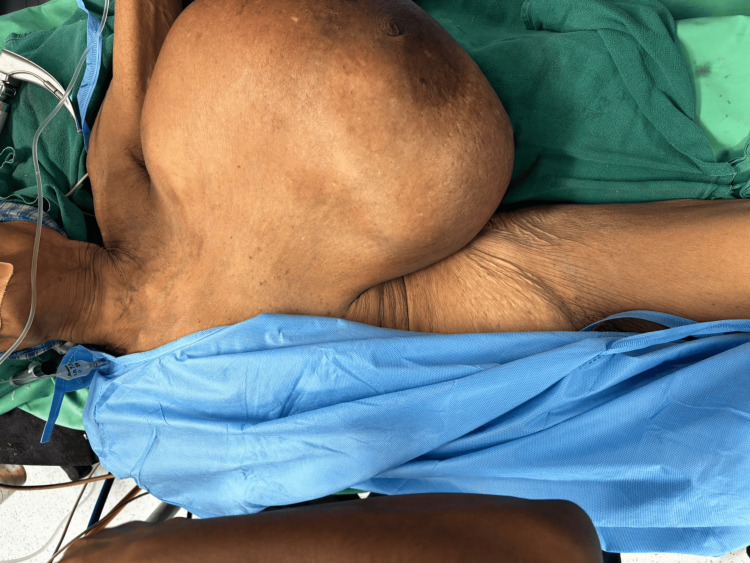
Preoperatively, the patient was positioned supine with the table tilted to her left to support the mass

**Figure 5:**
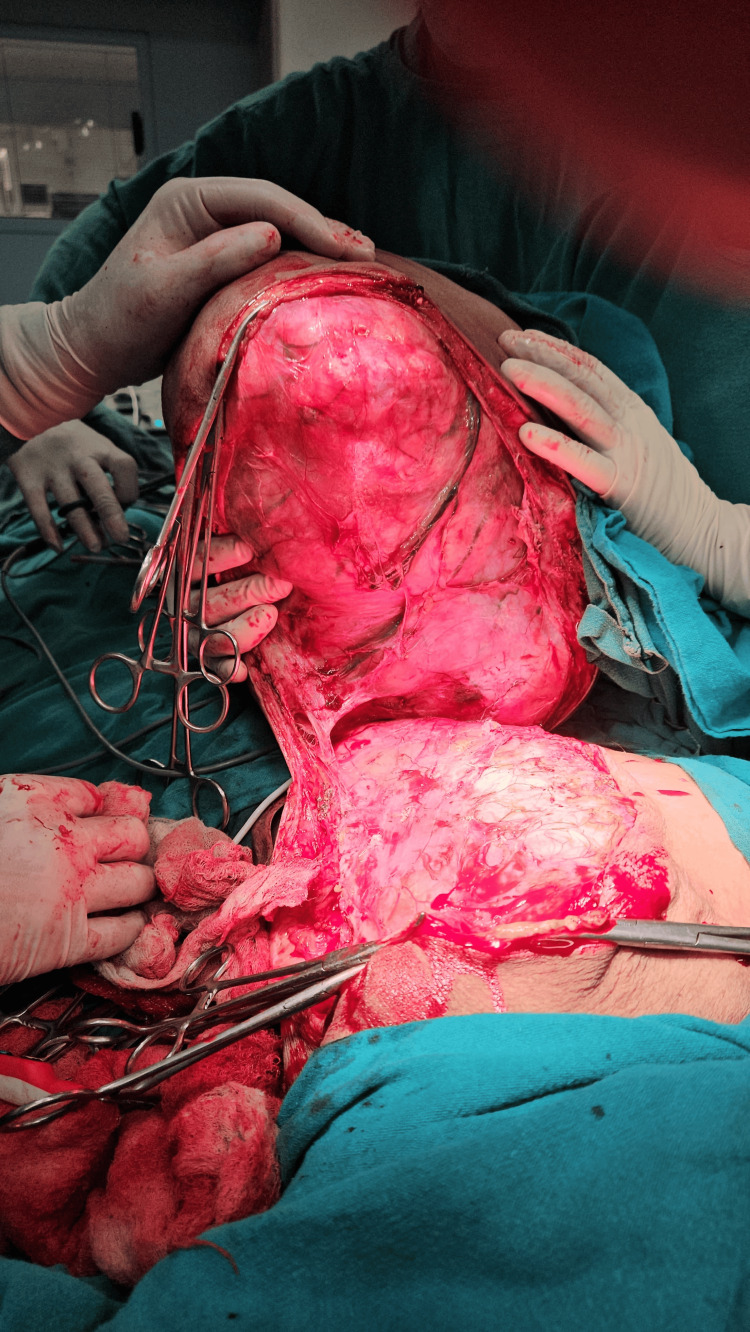
The tumor was lifted from the chest wall with fascia

**Figure 6:**
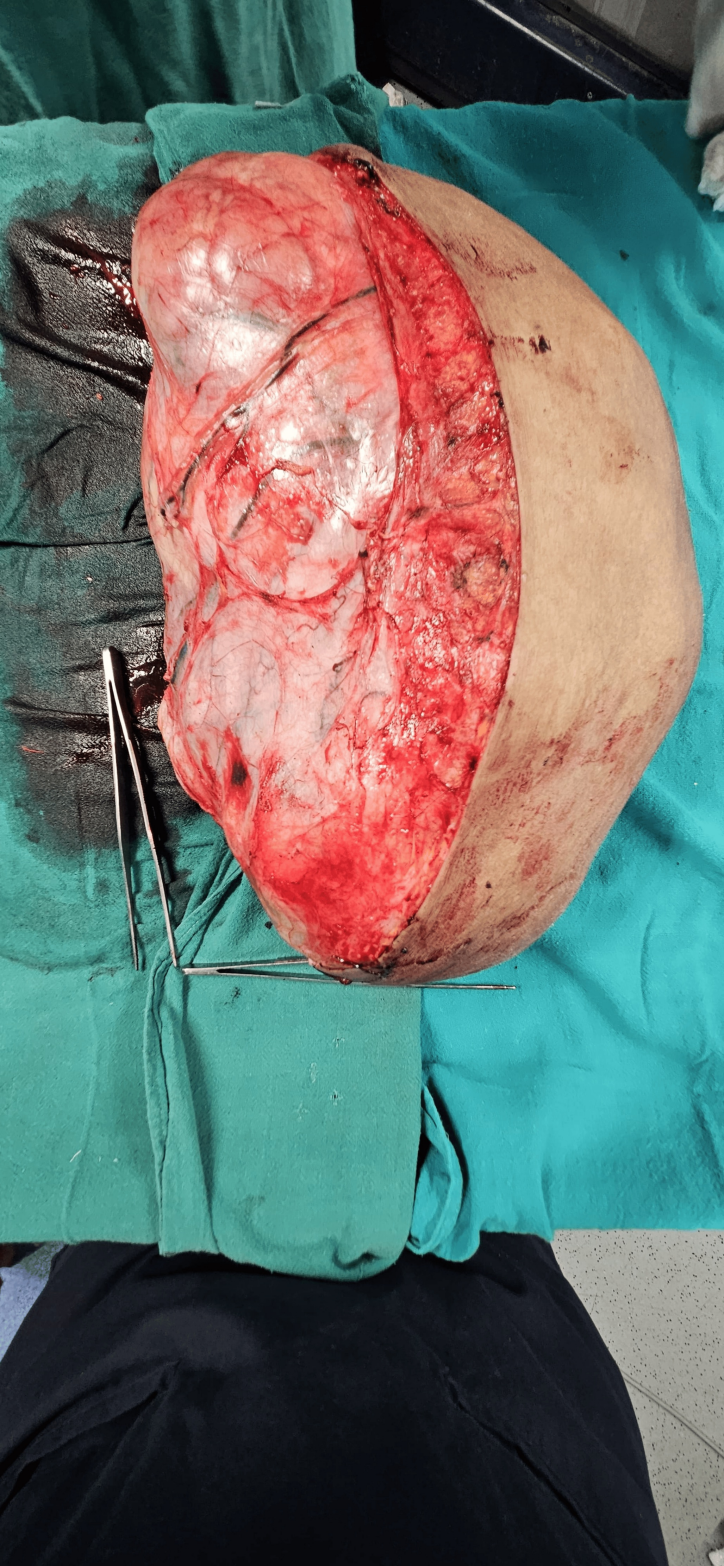
Tumor removed in toto with dimensions of 36x24x20 cm

**Figure 7:**
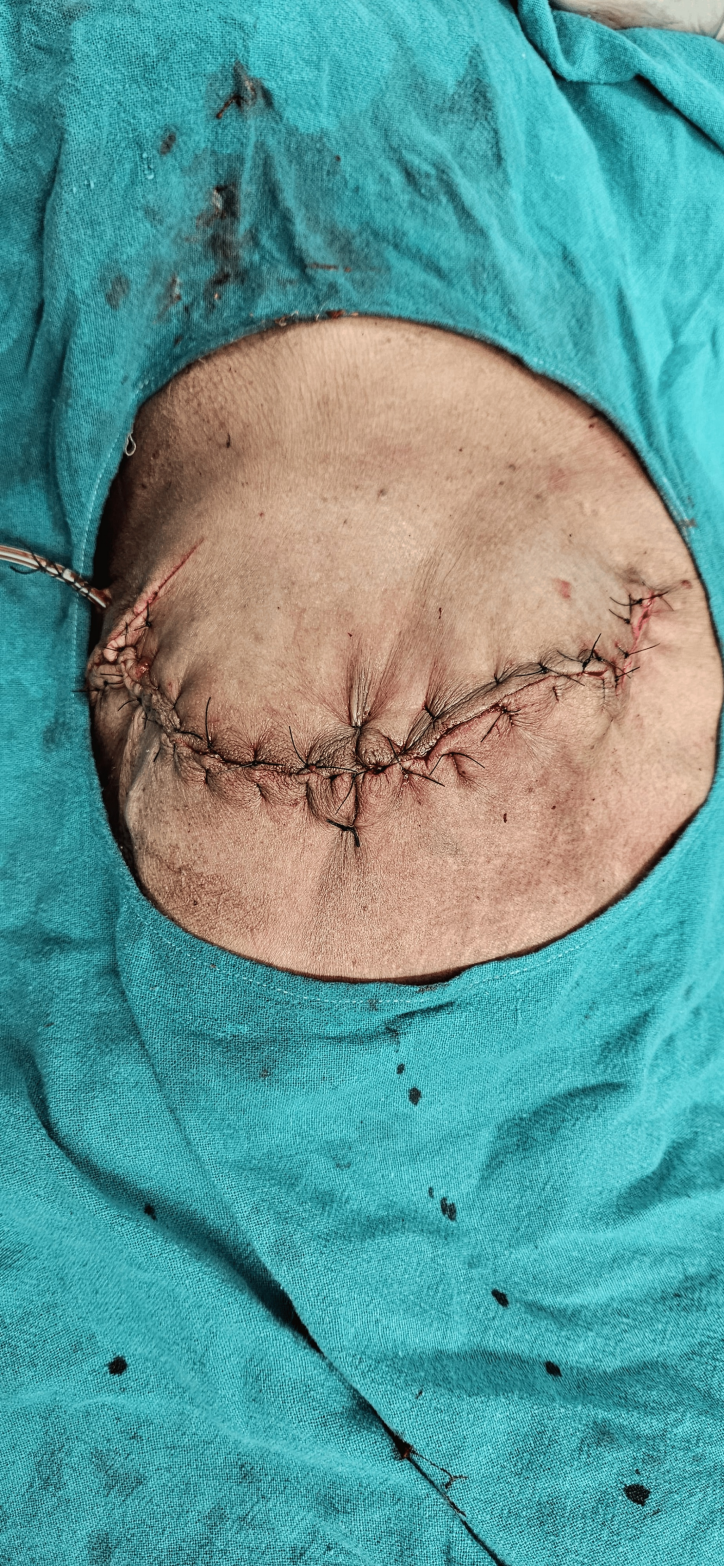
Primary skin closure was done after removing excess skin and a Romo Vac suction drain was placed

The patient was posted for surgery after optimization of abnormal parameters. A blood transfusion was done for the correction of anemia, and incentive spirometry was advised. The patient underwent a simple mastectomy after a detailed preanesthetic checkup and cardiac fitness. She was positioned in a supine position with a table tilted to her left to facilitate lung expansion during surgery (Figure 4). A circumferential incision was taken under full aseptic precaution. A surgical plan was identified after careful dissection. Feeding vessels were identified, ligated, and cut to minimize blood loss. The tumor was lifted from the chest wall and removed in toto with intact fascia (Figure 5 and Figure 6). The extra skin was removed and closed after placing a closed suction drain (Figure 7). The post-operative period was uneventful, and the patient was discharged on postoperative day 8.

**Figure 8:**
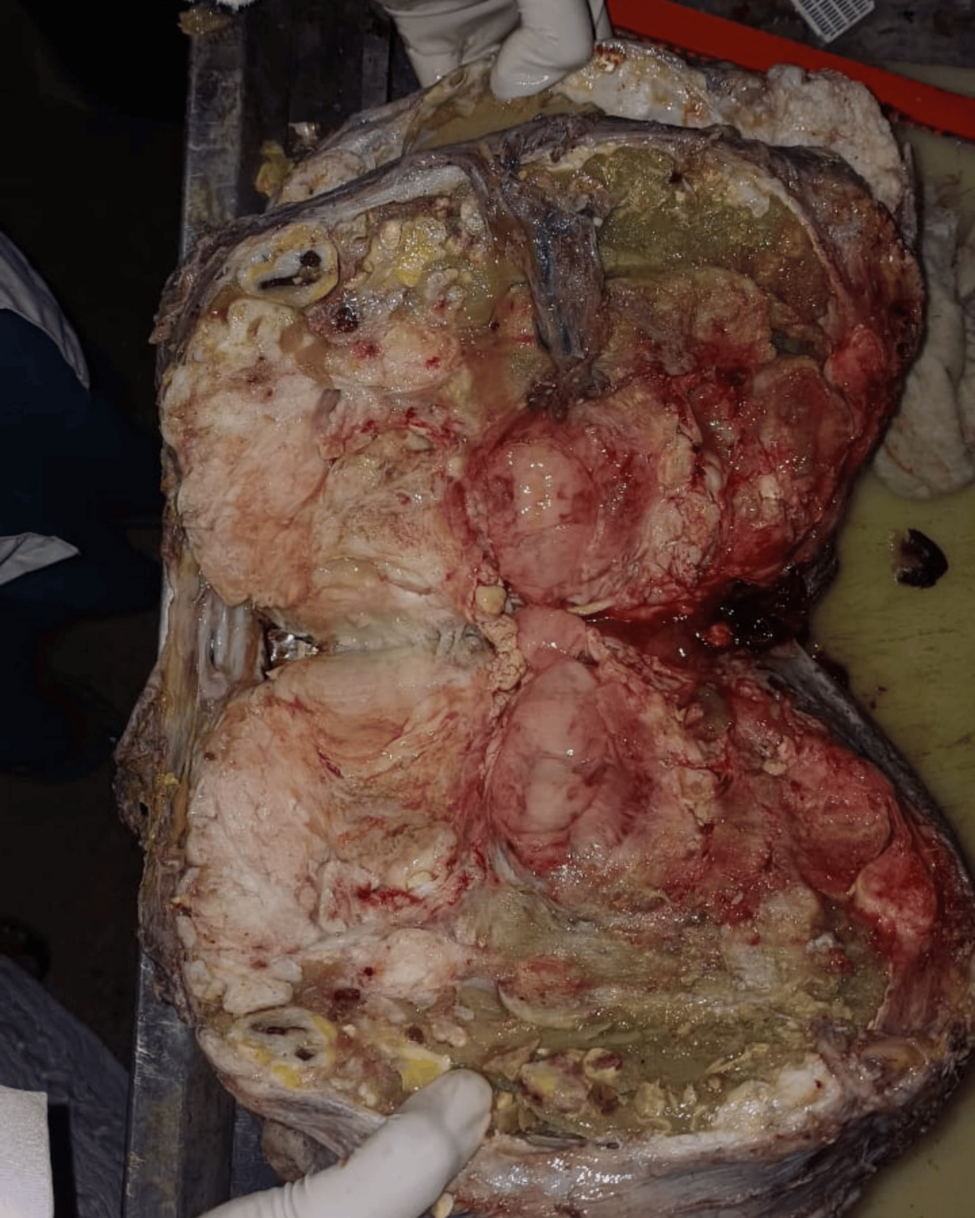
Cut section of the tumor with both cystic and solid components and varied degrees of calcification

**Figure 9:**
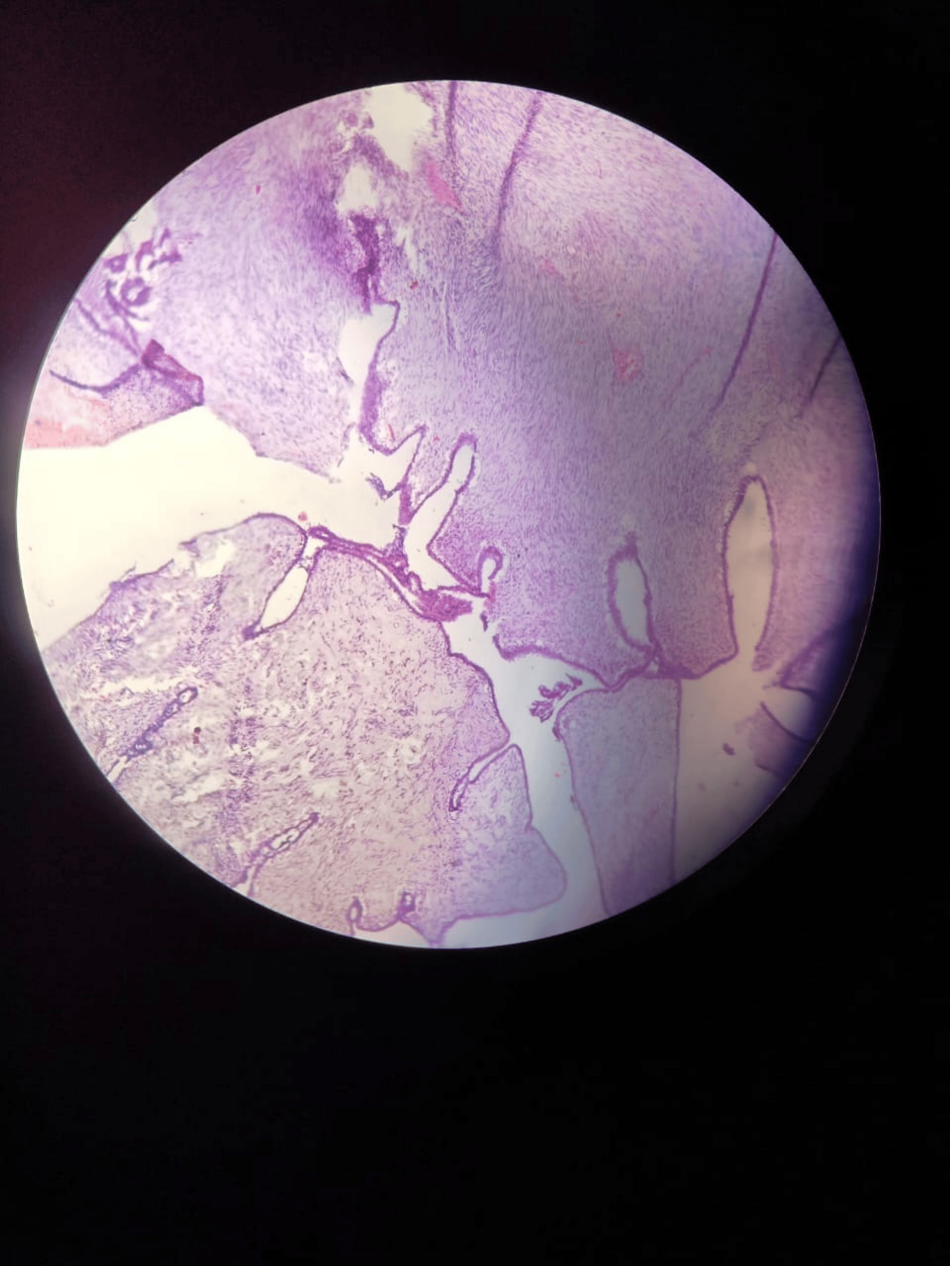
Histopathological examination revealed a markedly hypercellular stroma with mild atypia and a mitotic rate of 2/10 high-power fields, consistent with a benign phyllodes tumor

The tumor weighed 10.5 kg on the weighing scale. On the cut section, the mass showed a fleshy cream-colored inner section with multiple cystic and solid components (Figure 8). The mass was sent for histopathological examination (Figure 9). Her back pain was immediately relieved. However, postural deformity persisted. The patient was advised physiotherapy and a posture corrector brace.

## Discussion

Phyllodes have presented a great deal of dilemmas for surgeons and physicians for decades. Surgical management of the phyllodes tumor has been addressed many times in the literature. Giant phyllodes tumors always possess several unique challenges varying from case to case. Most of them are benign and present at an early stage. Borderline and malignant phyllodes tumors tend to have a higher recurrence rate than benign ones. Kim et al. found that tumors less than 4 cm in size generally receive less aggressive management. Hence, they reoccur more often and earlier [10]. However, in another study, it was found that tumors larger than 4 cm had a high risk of recurrence. Larger tumor size, high mitotic index, stromal proliferation, malignant subtype, and cellular atypia are important risk factors for relapse of the tumor [11]. In our case, the woman had a giant phyllodes tumor of benign type, and the tumor was removed with adequate tumor margin. There was no sign of recurrence in the three-month follow-up of the patient with significant improvement in her symptoms.

## Conclusions

Phyllodes tumors can largely mimic breast carcinoma and require prompt clinical and histological diagnosis. Lack of awareness, absence of rural screening programs, and patient ignorance may lead to the presentation of phyllodes in such huge forms and pose difficulty in their management. Breast cancer awareness programs should be done at primary health care centers. Promotion of self-breast examination (SBE) and education regarding danger signs of breast lump should be provided by peripheral health care workers.
